# Classification of anticancer drugs: an update with FDA- and EMA-approved drugs

**DOI:** 10.1007/s10555-024-10188-5

**Published:** 2024-07-05

**Authors:** Lorena Ostios-Garcia, Daniel Martínez Pérez, Beatriz Castelo, Noelia Hernández Herradón, Pilar Zamora, Jaime Feliu, Enrique Espinosa

**Affiliations:** 1 Department of Medical Oncology, Hospital Universitario La Paz, Madrid, Spain; 2 Universidad Autónoma de Madrid, School of Medicine - Department of Medical Oncology, Hospital Universitario La Paz, Madrid - CIBERONC, Madrid, Spain

**Keywords:** Antineoplastic agents, Classification, Chemotherapy, Tyrosine-kinase inhibitors, Monoclonal antibodies, Immunotherapy

## Abstract

Anticancer systemic therapy comprises a complex and growing group of drugs. Some of the new agents with novel mechanisms of action that have appeared are difficult to fit in the groups of classical chemotherapy, hormones, tyrosine-kinase inhibitors, and monoclonal antibodies. We propose a classification based on two levels of information: the site of action and the mechanism of action. Regarding the former, drugs can exert their action in the tumor cell, the tumor vasculature, the immune system, or the endocrine system. The mechanism of action refers to the molecular target.

## Introduction

Cancer encompasses a heterogeneous set of diseases with specific etiology, natural history, prognosis, and treatments, causing approximately 10 million deaths worldwide each year [1]. For this reason, new effective drugs are needed and proper understanding of the characteristics of available treatments is essential. Nitrogen mustard was the first drug to show tumor regression in patients with Hodgkin lymphoma [2]. In addition, it was the first chemotherapy drug approved by the US Food and Drug Administration (FDA) for human use in 1949 [2]. Since then, new agents have been incorporated into the clinic, so that over 100 are currently available.

Traditionally, agents have been grouped as chemotherapy, hormonal therapy, and immunotherapy. In the last 20 years, immunoconjugates, adoptive immunotherapies, tyrosine-kinase inhibitors, and other targeted therapies have been added to the list. There is a wide variety of mechanisms of action that cannot be included in a simple classification. The main goal of this paper is to establish an intuitive, useful, and comprehensive classification of classical and new cancer drugs. A classification serves two main objectives: the achievement of a comprehensive view of the available drugs and the design of combination therapy or new pharmacological strategies. A global view also serves teaching purposes.

## General concepts and classification

The classification includes agents approved by either the FDA or the European Medicine Agency (EMA) until March 2024. The approval year has been included next to each drug in the tables. We have excluded FDA-authorized drugs through the accelerated approval mechanism, and EMA conditionally approved drugs, both of which require further data in confirmatory trials demonstrating clinical benefit. In the case of drugs with more than one mechanism of action, the main one has been selected for our classification. In the case of immunoconjugates, which comprises a monoclonal antibody joined to a cell-killing substance, the payload has been considered for the classification. The mechanism of action and toxicity of every group are briefly described, although it is beyond our scope to discuss them in detail.

**Fig. 1:**
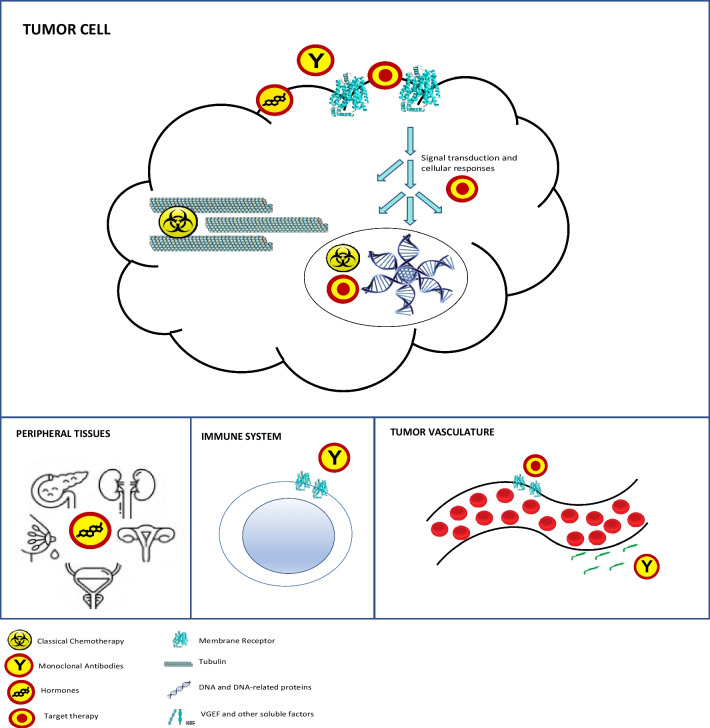
Sites and mechanisms of action of anticancer drugs. Drugs exert their action either on tumor cells, tumor vasculature, the immune system, or peripheral tissues

Antineoplastic agents can be first classified according to their therapeutic target and level of action (Fig. 1 and Table 1). Most of them directly interact with tumor cells, either in the nucleus, the cytoplasm, or the membrane. Others are aimed at the vascular endothelium or peripheral tissues, such as glands. Finally, a rapidly growing group of drugs target the immune system.

**Table 1:** Classification based on site of action

Target		Mechanism of action
Tumor cell	**Nucleus** (Table 2)	**Direct DNA damage**: alkylating agents **Disruption of DNA synthesis or RNA transcription:** antibiotics, antimetabolites **Inhibition of DNA-related proteins:** Camptothecin analogs, antibiotics, antimetabolites, deacetylase inhibitors, PARP inhibitors, HIF2 inhibitors, chaperone inhibitors, cyclin inhibitors **Other**: inhibition of XP01
	**Cytoplasm proteins** (Table 3)	**Tubulin destabilization or stabilization**: vinka alkaloids and taxanes **Inhibition of proteins in metabolic pathways:** *TKIs* of BRAF, BTK, KRAS, MEK, mTOR, NTRK, PI3K **Link to hormonal cytoplasm receptors:** antiestrogens, androgen receptor antagonists **Other:** inhibition of proteasome, inhibition of IDH
	**Membrane receptors** (Table 3)	**Inhibition of overexpressed or mutated receptors (ALK, CD20, CD38, EGFR, FGFR, HER2, Kit, Met, Nectin-4, NTRK, Ret):** immunoconjugates, monoclonal antibodies, TKIs
Vascular endothelium (Table 4)	**Membrane receptors**	VEGFR inhibitors
	**Soluble factors**	VEGF inhibitors
Immune system (Table 6)	**Membrane receptors and soluble factors of T lymphocytes**	**Inhibition of immune checkpoints (CTL4, PD1, PDL1, LAG3)**: monoclonal antibodies **T-cell engager**: - **Adoptive cell therapy**: CAR-T, TCR - **Bispecific molecules**: BiTE, immTAC
	**Immune-modulators**	**Link to cereblon**: thalidomide derivatives **Toll-like receptors agonists**: BCG and imiquimod
	**Other**	Cytokines, vaccines, oncolytic viruses
Endocrine System (Table 7)	**Estrogen-producing tissues**	Aromatase inhibitors
	**Hypophysis**	GnRH analogs and antagonists
	**Adrenal gland**	**17-Alpha-hydroxylase inhibitors**: abiraterone **Cytotoxic to adrenal cortex**: mitotane

*ALK*, anaplastic lymphoma kinase; *BCG*, bacillus Calmette-Guérin; *BiTE*, bispecific T-cell engager; *BTK*, Bruton’s tyrosine kinase; *CAR*, chimeric antigen-receptor; CTL4, cytotoxic T-lymphocyte-associated protein 4; *EGFR*, epidermal growth factor receptor; *FGFR*, fibroblast growth factor receptor; *GnRH*, gonadotropin-releasing hormone; *HER2*, human epidermal growth factor receptor 2; *HIF2*, hypoxia-induced factor 2; *IDH*, isocitrate dehydrogenase; *ImmTAC*, immune mobilizing T-cell receptors against cancer; *Kit*, receptor tyrosine kinase; *LAG3*, lymphocyte-associated gene 3; *MEK*, mitogen-activated protein kinase kinase; *Met*, receptor of the hepatocyte growth factor; *mTOR*, mammalian target of rapamycin; *NTRK*, neurotropic receptor tyrosine kinase; *PARP*, poly-ADP ribose polymerase; *PD1*, programmed death-1; *PDL1*, programmed cell death ligand 1; *PI3K*, phosphatidylinositol-3 kinase; *RET*, rearranged during transfection; *TKIs*, tyrosine kinase inhibitors; *VEGFR*, vascular endothelial growth factor; *TCR*, T cell receptor

A second level of classification pertains to the mechanism of action, as shown in Tables 2, 3, 4, 5 and 6. The mechanism of action refers to the molecular target, which makes sense because most agents block the activity of molecules and biological pathways that are required for tumor proliferation. Classical chemotherapy, hormone derivatives, tyrosine kinase inhibitors, and monoclonal antibodies appear scattered throughout the classification as they may have a variety of mechanisms of action.

**Table 2:** Drugs with direct activity against the nucleus of tumor cells

**Alkylating agents**	**Nitrogen mustards**: chlorambucil, cyclophosphamide, ifosfamide, melphalan, mecloretamina, bendabustine **Nitrosureas**: carmustine, lomustine, streptozocin **Platinum salts**: carboplatin, cisplatin, oxaliplatin **Triazenes**: dacarbazine, temozolomide **Other groups:** Ethylenimines: altretamine, thiotepa Hidrazine derivative: procarbazine Sulfonates: bendamustine, busulfan Ecteinascidin derivative: trabectedin
**Disruption of DNA or RNA synthesis**	**Antifolates**: methotrexate, pemetrexed **Thymidilate synthase inhibitors**: capecitabine, 5-fluorouracil, raltitrexed, TAS-102 **Purine analogs**: cladribine, fludarabine, mercaptopurine, pentostatin **Pyrimidide analogs**: cytarabine, gemcitabine **Ribonucleotide reductase inhibitors**: hydroxyurea **DNA-methyltransferase inhibitors**: azacytidine, decitabine Immunconjugates*: gentuzumab ozogamicina **Hydrolytic catalyzation**: l-asparaginase
**Inhibition of DNA-related proteins**	**Topoisomerase I inhibitors**: camptothecin analogs—irinotecan, topotecan Immunconjugates*: trastuzumab deruxtecan, sacituzumab govitecan **Topoisomerase II inhibitors**: etoposide, mitoxantrone, teniposide **Antibiotics**: anthracyclines (daunorubicin, doxorubicin, epirubicin, idarubicin, valrubicin), bleomycin, dactinomycin, mitomycin C **Histone deacetylase inhibitors**: belinostat, entinostat, vorinostat **PARP inhibitors**: niraparib, olaparib, rucaparib, talazoparib, veliparib **Hypoxia-inducible factor 2-alfa inhibitors**: belzutifan **Chaperone inhibitors**: Selinexor **Cyclin inhibitors**: abemaciclib, palbociclib, ribociclib
**Other mechanisms**	**Inhibition of XP01**: selinexor

*PARP*, poly-ADP ribose polymerase; *XPO1*, exportin

^*^This drug is classified according to its two components: cellular receptor ligand and payload

**Table 3:** Drugs with direct activity against proteins in the cytoplasm or the membrane of tumor cells

**Tubulin**	**Microtubule stabilization** Taxanes: cabazitaxel, docetaxel, paclitaxel, nab-paclitaxel Epothilone: ixabepilone Eribulin **Microtubule antagonists** Vinca alkaloids: vinblastine, vincristine, vinflunine, vinorelbine Estramustine Immunoconjugates*: trastuzumab-emtansine
**Intracellular metabolic pathways**	**Androgen receptor**: apalutamide, bicalutamide, enzalutamide, flutamide **Selective estrogen receptor modulator** (SERM): tamoxifen **Selective estrogen receptor degrader/downregulator** (SERD): fulvestrant **BRAF**: dabrafenib, encorafenib, vemurafenib **BTK**: ibrutinib **KRAS**: sotorasib **HRAS**: tipifarnib **MEK**: binimetinib, cobimetinib, trametinib **mTOR**: sirolimus, everolimus, temsirolimus **PI3K**: alpelisib, idelalisib **Proteasome**: borterzomib, carfilzomib, ixazomib **IDH**: ivosidenib, enasidenib
**Overexpressed or mutated membrane receptors**	**ALK**: alectinib, brigatinib, ceritinib, crizotinib **CD20**: rituximab **CD22**: Immunoconjugates*: inotuzumab ozogamicin **CD33**: Immunoconjugates*: gentuzumab ozogamicin **CD38**: isatuximab **EGFR**: TKIs: afatinib, erlotinib, gefitinib, osimertinib Monoclonal antibodies: cetuximab, panitumumab **FGFR2**: pemigatinib, infigratinib, futibatinib **HER2**: TKIs: lapatinib, neratinib, tucatinib Monoclonal antibodies: pertuzumab, trastuzumab Immunconjugates:* trastuzumab-emtansine, trastuzumab deruxtecan **Kit**: avapritinib, bosutinib, dasatinib, imatinib, ponatinib **Met**: pralsetinib, tepotinib **NTRK**: entrectinib, larotrectinib **Ret**: selpercatinib **Trop 2**: Immunoconjugates*: sacituzumab govitecan **SSTRs**: octreotide, lanreotide

*ALK*, anaplastic lymphoma kinase; *BTK*, Bruton’s tyrosine kinase; *EGFR*, epidermal growth factor receptor; *FGFR2*, fibroblast growth factor receptor 2; *HER2*, human epidermal growth factor receptor 2; *IDH*, isocitrate dehydrogenase; *Kit*, receptor tyrosine kinase; *MEK*, mitogen-activated protein kinase kinase; *Met*, mesenchymal epithelial transition factor receptor; *mTOR*, mammalian target of rapamycin; *NTRK*, neurotrophic tyrosine receptor kinase; *PI3K*, phosphatidylinositol-3 kinase; *Ret*, rearranged during transfection; *SERM*, selective estrogen receptor modulator; *TKIs*, tyrosine kinase inhibitors

^*^This drug is classified according to its two components: cellular receptor ligand and payload

**Table 4:** Drugs with activity in the tumor vasculature

**Membrane receptors**	**VEGFR** TKIs: axitinib, cabozantinib, lenvatinib, pazopanib, sunitinib, regorafenib, tivozanib, vandetanib, sorafenib Monoclonal antibodies: ramucirumab
**Soluble factors**	**VEGF**: Monoclonal antibodies: bevacizuma, aflibercept

*TKIs*, tyrosine kinase inhibitors; *VEGF*, vascular endothelial growth factor; *VEGFR*, vascular endothelial growth factor receptor

**Table 5:** Targets of multiple kinase inhibitors

	AXL	CSFR	FGFR	FLT3	KIT	MET	PDGFR	RAF	RET	TIE2	VEGFR
Axitinib											X
Cabozantinib	X			X	X	X			X	X	X
Lenvatinib			X		X		X		X		X
Pazopanib					X		X				X
Sorafenib							X	X			X
Sunitinib			X	X	X		X	X			X
Regorafenib		X	X				X	X	X	X	X
Tivozanib					X						X
Vandetanib									X		X

*CSFR*, granulocyte colony-stimulating factor receptor; *FGFR*, fibroblast growth factor receptor 2; *FLT3*, fms-like tyrosine kinase 3; *KIT*, receptor tyrosine kinase; *MET*, mesenchymal epithelial transition factor receptor; *PDGFR*, platelet-derived growth factor receptor; *RAF*, rapidly accelerated fibrosarcoma; *RET*, rearranged during transfection; *TIE2*, tyrosine kinase with immunoglobin and EGF homology domains; *VEGFR*, vascular endothelial growth factor receptor

**Table 6:** Drugs with activity in the immune system

**Membrane receptors of T lymphocytes and soluble factors**	**Immune checkpoint inhibitors** PD1: dostarlimab, nivolumab, pembrolizumab PD-L1: avelumab, atezolizumab, durvalumab CTLA-4: ipilimumab LAG3: relatlimab **T-cell engaging therapy**: Adoptive cell therapy: CAR-T: axicabtagene ciloleucel, tisagentecleucel, brexucabtagene autoleucel, ciltacabtagene autoleucel, lisocabtagene maraleucel, idecabtagene vicleucel Bispecific molecules: BiTE: blinatumomab ImmTAC: tebentafusp
**Immune modulators**	**Link to cereblon**: lenalidomide, pomalidomide, thalidomide BCG Imiquimod
**Other**	**Cytokines**: interferon, interleukin 2 **Oncolytic viruses**: talimogene-laherparepvec (T-VEC) **Vaccines**: sipuleucel

*BiTE*, bispecific T-cell engager; *CAR*, chimeric antigen-receptor; *CTL4*, cytotoxic T-lymphocyte-associated protein 4; *ImmTAC*, immune mobilizing T-cell receptors against cancer; *LAG3*, lymphocyte-associated gene 3; *PD1*, programmed death-1; *PDL1*, programmed cell death ligand 1; *T-VEC*, talimogene laherparepvec

## Drugs directed against tumor cells

Drugs in this group can exert their action in the nucleus, in the cytoplasm or in membrane receptors.

### Drugs with a direct action in the nucleus of tumor cells (Table 2)

Deoxyribonucleic acid (DNA) was identified as a useful target many decades ago, so most agents of classical chemotherapy belong to this group. Alkylating agents (subclassified as nitrogen mustards, platinum salts, nitrosoureas, triazanes, and methylating agents) alkylate nucleic acids and proteins. This creates cross-links that disrupt and break DNA during duplication, inducing cell cycle arrest and apoptosis [3]. Alkylating agents are used for the treatment of a wide variety of hematological and solid tumors. Major toxicities include nausea and vomiting, myelosuppression, alopecia, infertility, and teratogenesis [3].

Antimetabolites are structural analogs of purine or pyrimidine bases, which are involved in purine and pyrimidine biosynthesis, or of folate cofactors, which are responsible for the inhibition of ribo- and deoxyribonucleotide synthesis [3]. They compete with normal metabolites (leading to aberrant DNA and RNA synthesis) or interfere with enzymes involved in the production of nitrogenous bases. DNA polymerase, ribonucleotide reductase inhibitors, DNA methyltransferase inhibitors, and other drugs with non-specific mechanisms of action are also considered antimetabolites. These agents are used to treat a variety of hematological tumors and some solid tumors such as breast or digestive carcinomas. The main side effects of these agents are myelosuppression and gastrointestinal [3].

DNA replication requires the DNA helix to be separated, which results in torsional stress that is resolved by DNA topoisomerases I (TOP1) and II (TOP2) [3]. The inhibition of TOP1 and TOP2 induces single- and double-strand breaks of DNA stopping the cell cycle. Camptothecins inhibit TOP1 catalysis, whereas the anthracyclines (a group of antibiotics) and epipodophyllotoxins target TOP2 [3]. Other antibiotics different from anthracyclines also prevent DNA replication by interfering with topoisomerases or inducing DNA breaks or DNA cross-links.

The immunoconjugate sacituzumab govitecan, that links to cells expressing the transmembrane glycoprotein Trop-2, is included among campthothecins, because its cytotoxic effect is mediated by the payload of the topoisomerase inhibitor SN38.

These drugs can be used to treat different solid tumors and lymphomas. Major toxicities of topoisomerase inhibitors are vomiting, myelosuppression, alopecia, and, in the case of anthracyclines, also cardiotoxicity [3].

Other agents target DNA-related proteins such poly-ADP polymerase (PARP), histone deacetylase, chaperones, or cyclin-dependent kinases (CDK) 4/6 [3, 4]. PARP is an enzyme involved in DNA transcription and repair, and its inhibition is useful to treat tumors with mutations in BRCA1 (breast cancer gene 1) and BRCA2 (breast cancer gene 2) and other genes involved in the repair of homologous recombination deficits. PARP inhibitors have been approved for the treatment ovarian, breast, and pancreatic cancer [5]. CDKs have an important role in regulating cell cycle progression. Alterations in the CDK4/6 pathway are associated with endocrine resistance in breast cancer [6], so CDK 4/6 inhibitors [4] act synergistically with hormonal therapies [6].

### Drugs aimed at the cytoplasm of tumor cells (Table 3)

Tubulin-targeting drugs are plant-derived agents that prevent the formation of microtubules and induce cell cycle arrest. This category contains taxanes and epothilones, which stabilize microtubules and avoid their disassembly, and vinca alkaloids, which bind the tubulin of microtubules inhibiting their assembly [3]. Nab-paclitaxel is a formulation of paclitaxel bound to albumin, which activates transcytosis and allows a higher concentration of the drug inside tumor cells [7].

Immunoconjugates have been recently incorporated to this group [8]. Brentuximab vedotin and enfortumab vedotin link to CD30 and nectin-4, respectively, but both membrane receptors simply serve to selectively internalize a payload of auristatin, a microtubule-disrupting agent [9]. In the case of trastuzumab-emtansine, also known as T-DM1, the cytotoxic agent is a maytansine derivative [9]. Nowadays, natural and semi-synthetic tubulin-targeting drugs are available for the treatment of solid tumors, including breast, lung, ovarian and prostate cancers, and leukemia and lymphoma. Neurotoxicity is a common side effect of all these compounds, along with myelosuppression [3].

Besides from drugs interacting with microtubules, others have been incorporated in recent years that target proteins in the cytoplasm of tumor cells, more specifically those directed against tyrosine kinases of metabolic pathways [10, 11]. They are also known as small molecules or targeted therapy, but such denominations do not reflect their mechanism of action and refer to features shared with other anticancer drugs. Tyrosine kinase therapy is based on the principle that some malignancies take advantage of specific metabolic pathways to proliferate and survive. One of more important pathways is the mitogen-activated protein kinases (MAPK) cascade, a family of intracellular protein kinases (RAS/RAF/MEK/ERK) that regulate many aspects of cell functions such as growth, survival, differentiation, and cellular communication [12]. Imatinib, an inhibitor of the ABL kinase, paved the way for drugs inhibiting many other cytoplasmic proteins, such as those in the MAPK and phosphatidylinositol-3 kinase (PI3K) pathways [13]. Tyrosine kinase inhibitors are used to treat solid and hematological tumors. The toxicity of these drugs varies with the specific target, although fatigue, skin, and digestive side effects are common [3].

Proteasome inhibitors are a group of anticancer drugs which inhibit the catalytic active subunits of the proteasome, a multicatalytic protein complex that degrades many cellular proteins [14]. Proteasome inhibitors affect protein turnover, which is ultimately toxic to the cells [14]. They are used to treat multiple myeloma and mantle cell lymphoma.

Although hormonal therapy usually exerts its action on endocrine glands, selective estrogen receptor modulators (SERM) such as tamoxifen, and selective estrogen receptor degrader (SERD) such as fulvestrant, mainly target proteins located within the tumor cell cytoplasm [15, 16]. SERMs bind to intracellular estrogen receptor (ER) competing against estrogen. ER-SERM complexes are transferred to nucleus of tumor cells, where they interact with transcriptional factors [17]. SERDs create a protein complex with estrogen receptor that induces degradation via the proteasome [17]. SERM and SERD are approved for treatment of breast cancer.

### Drugs aimed at membrane receptors of tumor cells (Table 3)

Many membrane receptors couple to growth factors to initiate a signaling cascade that promotes proliferation [18]. Drugs blocking membrane receptors may be monoclonal antibodies or tyrosine kinase inhibitors. When receptor tyrosine kinases are overexpressed by gene amplification or transcriptional/translational enhancement, aberrant downstream signal transduction follows [19]. Antibodies are large molecules that do not enter tumor cells, so they interact with the external domain of the receptors [20]. Tyrosine kinase inhibitors, on the other hand, cross the cell membrane and interact with the intracellular domain of the receptor [11].

Drugs targeting epidermal growth factor receptors (either EGFR or HER2) or CD20 are widely used in the clinic, whereas those aimed at anaplastic lymphoma kinase (ALK), fibroblast growth factor receptor, Kit, or neurotrophic tyrosine receptor kinase (NTRK) are useful in a minority of tumors with very specific mutations or translocations. Toxicity varies widely depending on the specific target.

Neuroendocrine tumors express G-protein-coupled transmembrane somatostatin receptors (SSTRs) [21]. Somatostatin analogs such as octreotide and lanreotide bind to SSTR and produce a wide variety of effects, including decreased hormone secretion, decreased growth, and proliferation and increased apoptosis [21]. Somatostatin analogs are used in the treatment of neuroendocrine tumors [21].

## Drugs with activity in the tumor vasculature (Tables 4 and 5)

Most agents in this group are small molecules that target multiple tyrosine kinases. They have been classified together as anti-angiogenic drugs because they impair the blood vessels tumors that tumors require for sustained growth. They may also have a direct effect on tumor cells that share specific membrane receptors. Targets for this family of drugs are membrane receptors such as the fibroblast growth factor receptor (FGFR) and vascular endothelium growth factor receptor (VEGFR), intracellular kinases in tumor cells such as RET, or the vascular endothelial growth factor (VEGF) itself [10, 11, 22].

Anti-angiogenic agents are used to treat renal cell carcinoma and other solid tumors. Inhibition of VEGFR accounts for the risk of hypertension [3]. Other common side effects are cutaneous and digestive [3].

## Drugs with activity in the immune system (Table 6)

Immunotherapy has revolutionized the treatment of cancer in recent years. The two main groups of drugs in this family are immune checkpoint inhibitors and T-cell engaging therapies. The targets of both types of agents may be present in tumor cells, but participation of the immune system is essential to their activity. Other groups considered as immunotherapy include immune modulators, cytokines, vaccines, and oncolytic viruses.

Immune check point inhibitors block membrane receptors of T lymphocytes or soluble factor that inactivate effector T-cells: programmed death-1 (PD-1), programmed death ligand 1 (PD-L1), cytotoxic T-lymphocyte-associated antigen 4 (CTLA4), and lymphocyte-activation gene 3 (LAG3) [23, 24], among others. These checkpoints limit the activity of effector T-cells, so blocking antibodies boost the immune response against cancer cells. It is also possible to use antibodies to activate check points that enhance the activity T-cells, but these are still under development. Toxicity of check point inhibitors is mediated through infiltration by over-stimulated lymphocytes. It may consist of rash, colitis, hepatitis, pneumonitis, or thyroiditis, although any organ can be affected [23, 24].

T-cell engaging therapy comprises adoptive cell therapy and bispecific molecules [25, 26]. Chimeric antigen-receptor T (CAR-T) cells are a way of adoptive therapy: T-cells from the patient are collected and re-engineered to produce proteins on their surface called chimeric antigen receptors. CARs recognize and bind to specific proteins or antigens of cancer cells [27, 28]. Tumor-infiltrating lymphocytes (TILs) are another type of adoptive cell therapy in which lymphocytes already conditioned to the tumor microenvironment are harvested and subjected to chemokine fortification prior to reintroduction in the patient [29]. Bispecific T-cell engagers (BiTES) are bispecific molecules that bind to the CD3 subunit of T-cells and a tumor-associated antigen [26]. If this antigen is located on the tumor cell surface, tumor killing does not depend on presentation by the major histocompatibility complex (MHC) [25]. On the contrary, presentation by MHC (usually HLA-A*0201) is required in the case of intracellular tumor antigens [25, 30]. One way to target MHC-presented intracellular antigens is using engineered T-cell receptor peptides fused to an anti-CD3, which is known as immune mobilizing monoclonal TCRs against cancer (ImmTACs) [30]. Cytokine-releasing syndrome is the most common toxicity of T cell receptor-engineered T cell (TCR-T), CAR-T cells, and bispecific molecules [25, 26].

Immunomodulators are a group of drugs with anti-angiogenic and anti-inflammatory activity that are widely used as a treatment of hematologic tumors [31]. The antitumor effect is related to (1) stimulation of the cellular immunity mediated by T lymphocytes [32, 33] and natural killer lymphocytes [34], (2) suppressive effect on proinflammatory cytokines such as tumor necrosis factor (TNF-α) or interleukin 6 [31], and (3) blocking of migration and adhesion of endothelial cells and the formation of micro vessels [31]. These drugs can cause side effects such as drowsiness, fatigue, constipation, low blood cell counts, thromboembolic events, and neuropathy [35].

## Drugs with activity in endocrine system (Table 7)

**Table 7:** Drugs with activity in the endocrine system

**Estrogen-producing tissues**: Aromatase inhibitors	Anastrozole, exemestane, letrozole
**Hypophysis**: GnRH analogs and antagonists	Agonist: goserelin, leuprolide, triptorelin Antagonist: degarelix Progestational agents: megestrol acetate
**Adrenal glands**	17-Alpha-hydroxylase inhibitors: abiraterone acetate Mitotane

*GnRH*, gonadotropin-releasing hormone

Most of the drugs consider hormone therapy act on peripheral tissues where blocks the synthesis of hormones or prevents the activation of hormone receptors, which trigger a signaling cascade that promotes the expression of proliferation genes [15, 16, 36]. They are used to slow the growth of certain tumors which normally grow in response to physiological endocrine signaling. The aromatase inhibitors block local estrogen synthesis in breast tissue and are used in estrogen-positive receptor breast cancer for postmenopausal women [37]. The gonadotropin-releasing hormone (GnRH) agonists and antagonists are peptide analogs of GnRH, which cause an inhibition of estrogen and androgen synthesis [36]. They are used mainly as androgen deprivation therapy for prostate cancer [36]. Abiraterone is a selective inhibitor of 17-alpha-hydroxylase (CYP17), an enzyme required for androgen synthesis in testicular, adrenal, and prostate tumor tissues [38]. Mitotane is a drug with cytotoxic and antisecretory effects on adrenal cells which induces cell death by inhibiting the synthesis of adrenocortical steroids. It is approved for metastatic and unresectable adrenocortical carcinoma [39].

## Mechanisms of resistance

Despite the overwhelming number of available antineoplastic drugs, cancer remains one of the leading causes of death in the world. Tumor heterogeneity and plasticity account for resistance, so many patients will have tumor progression as the best response or will progress after an initial response. In the first situation, known as primary resistance, endogenous characteristics are presented in cancer cells or microenvironment before drug exposition and provide tumors with advantages and mechanisms to survive [40]. The second, known as acquired resistance, appears after cancer treatment stress as an adaptative response from cells to survive and compromised treatment efficacy in initially sensitive tumors [40].

Cancer cell resistance can be due to many factors (genetic, epigenetic, and microenvironmental factors) [40, 41], which are not mutually exclusive [40], and they can appear sequentially or simultaneously [41].

Changes in the stroma and tumor microenvironment [42], local immunity [41], presence of cancer stem cells [43], inactivation by detoxification enzymes [44], mutations in membrane transporters to reduce intracellular drug accumulation, increased drug degradation due to altered expression of drug-metabolizing enzymes [45], enhancement in DNA repair [46, 47], and gene amplification [47] have been described in the case of classical chemotherapy. In the case of tyrosine-kinase inhibitors, mutations or amplifications in the target protein and activation of alternative pathways are the main reasons for resistance [48]. Hypoxia and autophagy are also known to contribute to drug resistance and reduced drug efficacy [49]. Additional mechanisms of resistance have been described for immunotherapy, which can be classified into insufficient generation of anti-tumor T cells, inadequate function of those T cells, and impaired T cell memory [50]. Among them, the most characteristic are aberrant expression of tumor antigens, alterations in antigen presentation pathways, secretion of immunosuppressive molecules and cytokines, and changes in the microbiome [51].

To overcome cancer resistance, several strategies are beginning to emerge. Combination therapy is the main strategy employed. Drugs with different mechanisms of action are given concurrently to produce complementary or synergistic effect and increase cell killing with minimally overlapping toxicities. In the case of tyrosine kinase inhibitors, new generation compounds can tackle a broader range of mutations. In the future, the identification of targetable, heterogeneous mechanisms of drug resistance will require to conduct sequential tumor biopsies during treatment: samples will be analyzed with the use of new technologies to identify mechanisms of resistance and to design a personalized strategy in every patient [40].

## Trends in the development of anticancer drugs

In the twentieth century, classical chemotherapy and hormones dominated the landscape of anticancer drugs. Better knowledge of the molecular biology of cancer led to the era of therapies targeting tyrosine kinases in the early twenty-first century. In the last 10–15 years, while new targeted therapies have appeared, immunotherapy has emerged as an option for many tumor types.

In this context, therapies based on adoptive treatments with T cells stand out, such as tumor-infiltrating lymphocytes (TILs), T cells with chimeric antigen receptor (CAR), and bispecific antibodies, as well as cancer vaccines are currently being investigated. However, some factors limit its development, especially in solid tumors. Among these factors, we highlight the heterogeneity of the tumors, the immunosuppressive microenvironment, the presence of toxicities, and the high cost associated with their production [52]. CAR-NK have also demonstrated their effectiveness in clinical studies, where they appear to be a more effective alternative to CART as they are independent of the tumor antigen, and without presenting side effects such as the release of cytokines and neurotoxicity. Gene editing with CRISPR technology could offer a therapeutic alternative in the future by editing or repairing defective genes related to the appearance and progression of cancer or genetically redirecting T cells to mutant neoantigens [53, 54]. Meanwhile, immunoconjugates and antibody–drug conjugates (ADCs) are considered the beginning of a new era in cancer treatment [52].

Finally, in the era of artificial intelligence (AI), profiling based on genetic and molecular characteristics using AI could help predict the therapeutic approach or drug combination that best suits each tumor and patient.

## Conclusions

Drug development in oncology has increased significantly in the last decades and there is a wide variety of drugs with complex mechanisms of action. In this review, we propose a classification that firstly considers the site of action and then the mechanism of action. This would allow to easily classify new agents in the future.
